# Population Pharmacokinetic/Pharmacodynamic Modelling of Daptomycin for Schedule Optimization in Patients with Renal Impairment

**DOI:** 10.3390/pharmaceutics14102226

**Published:** 2022-10-18

**Authors:** Teresa García-Martínez, María Dolores Bellés-Medall, Maria García-Cremades, Raúl Ferrando-Piqueres, Victor Mangas-Sanjuán, Matilde Merino-Sanjuan

**Affiliations:** 1 Department of Pharmacy and Pharmaceutical Technology and Parasitology, University of Valencia, 46100 Valencia, Spain; 2 Department of Pharmacy, University Hospital of Castellon, 12004 Castellon, Spain; 3 Department of Pharmaceutics and Food Technology, School of Pharmacy, Complutense University of Madrid, 28040 Madrid, Spain; 4 Interuniversity Research Institute for Molecular Recognition and Technological Development, 46022 Valencia, Spain

**Keywords:** daptomycin, pharmacokinetic, AUC, optimal dose selection, creatinine clearance

## Abstract

The aims of this study are (i) to develop a population pharmacokinetic/pharmacodynamic model of daptomycin in patients with normal and impaired renal function, and (ii) to establish the optimal dose recommendation of daptomycin in clinical practice. Several structural PK models including linear and non-linear binding kinetics were evaluated. Monte Carlo simulations were conducted with a fixed combination of creatinine clearance (30–90 mL/min/1.73 m^2^) and body weight (50–100 kg). The final dataset included 46 patients and 157 daptomycin observations. A two-compartment model with first-order peripheral distribution and elimination kinetics assuming non-linear protein-binding kinetics was selected. The bactericidal effect for Gram+ strains with MIC ≤ 0.5 mg/L could be achieved with 5–12 mg/kg daily daptomycin based on body weight and renal function. The administration of 10–17 mg/kg q48 h daptomycin allows to achieve bactericidal effect for Gram+ strains with MIC ≤ 1 mg/L. Four PK samples were selected as the optimal sampling strategy for an accurate AUC estimation. A quantitative framework has served to characterize the non-linear binding kinetics of daptomycin in patients with normal and impaired renal function. The impact of different dosing regimens on the efficacy and safety outcomes of daptomycin treatment based on the unbound exposure of daptomycin and individual patient characteristics has been evaluated.

## 1. Introduction

The clinical use of daptomycin in adult patients includes complicated skin and soft-tissue infections, right-sided infective endocarditis and bacteriemia due to Staphylococcus aureus (*S. aureus*), with an approved posology ranging from 4 to 6 mg/kg once daily for 7–14 days or until the resolution of the infection [1,2,3,4,5,6].The emergence of more resistant Gram-positive strains is leading to the recommendation and use of daptomycin at higher than approved dosage levels [7,8,9,10,11,12,13,14,15], especially in critically ill patients, which increases the uncertainty of clinicians when managing patients who also present renal-type disorders or extreme weight values.

The pharmacokinetic (PK) properties of daptomycin have been investigated in multiple publications in recent years, both in adult and pediatric patients [16,17,18]. So far, linear PK processes of daptomycin have been proposed, which show a rapid disposition phase followed by a slower and sustained disposition phase over time. The excretion of daptomycin is primarily promoted by the kidneys, with 54–78% eliminated in urine and a plasma elimination half-life from 7.6 to 8.8 h [3,5,6]. In addition, daptomycin is highly bound to plasma proteins, with a free fraction being around 10%. However, the pathophysiological alterations and clinical situation of patients may alter the protein binding kinetics [3,5], conditioning the time course of daptomycin in plasma and at the target site. It is essential that these elements be carefully considered to establish optimal dosage schemes based on the type of resistant microorganism, since in most cases the PK/PD efficacy indices are based on the total daptomycin values.

For all this, the development of model-informed strategies for the optimization of daptomycin treatment in patients is a fundamental tool in clinical practice. Creatinine clearance, albumin or body weight have been identified as clinically relevant covariates in the PK properties of daptomycin [17,19,20,21,22]. However, the use of therapeutic drug monitoring for the selection of an individualized daptomycin schedule could be additionally recommended [23], due to the moderate inter-individual variability on the PK parameters and the complex efficacy/safety balance. In this sense, Monte Carlo simulations represent a reliable and powerful methodology, which contributes to an optimal dose selection by integrating the variability in the PK parameters [20]. Recently, several authors have conducted a simulation-based analysis to suggest higher dose levels of daptomycin than approved to achieve bactericidal effects in adults [11,24,25,26] and pediatric [27,28] patients, especially on methicillin-resistant Staphylococcus aureus (MRSA) bacteremia. However, these studies include a narrow distribution of body weight and renal function conditions that impede a precise and specific evaluation of those effects on efficacy and safety outcomes. Therefore, the aims of the current work are (i) to develop a population pharmacokinetic/pharmacodynamic (PK/PD) model of daptomycin in patients with normal and impaired renal function, and (ii) to establish the optimal dose recommendation of daptomycin in clinical practice.

## 2. Materials and Methods

A prospective multi-center study in patients admitted to the General University Hospital of Castellón (Castellón, Spain) and the Arnau de Vilanova Hospital of Valencia (Valencia, Spain) was conducted. Patients who received daptomycin for any diagnosis were enrolled in the present study according to the following inclusion criteria: signed informed consent; administration daptomycin >48 h; and age >18 years. The exclusion criteria were patients with renal replacement therapy.

Daptomycin was administered by a 30-min intravenous infusion every 24 h, with daily doses ranging from 4 mg/kg up to 12 mg/kg by clinical practice.

Five serial blood samples were collected from a peripheral vein from day 4 (steady state) at the pre-dose, 0.5, 1–2, 4–10 h after the infusion ended and before the next dose. According to a TDM protocol for daptomycin adopted in these hospitals, the samples, collected in an additive-free tube, were centrifuged and the serum was stored at −20 °C until the drug concentrations were measured [29].

Demographic (age, body weight and height), treatment (dose and co-administered medications) and clinical data (microbiological data, analytical and disease covariates) were collected at patient enrolment. Serum creatinine and serum albumin were measured on the day (if not available, the nearest day) of measurement of the drug concentration. MIC were determined in all patients by e-test, but in patients where MIC were not available, the majority of the local MIC distribution to daptomycin was used (0.25 mg/L).

Creatinine clearance (CLCR) was estimated using the Cockroft-Gault formula [30] and normal renal function (CLCR: >90 mL/min/1.73 m^2^), mild renal impairment (CLCR: 60–90 mL/min/1.73 m^2^), moderate renal impairment (CLCR: 30–60 mL/min/1.73 m^2^), and severe renal impairment (CLCR: <30 mL/min/1.73 m^2^) were established.

Daptomycin plasma total concentrations were determined by a validated high-performance liquid chromatography assay with UV detection, which was available at our hospital center [31]. Precision and accuracy were assessed by performing replicate analysis of quality control samples against calibration standards. Intra- and inter-assay coefficients of the variation were always <10%. The lower limit of detection was 0.027 mg/L. Although free daptomycin fractions have been determined by ultra-performance liquid chromatography coupled to tandem mass spectrometry [32] (UPLC-MS/MS), this equipment was not available at our center and, therefore, no free daptomycin concentrations could be measured.

All data analyses were performed based on the population approach with the software NONMEM^®^ (Version 7.4; Icon Inc, PA, USA). The population PK parameters were estimated using the Stochastic Approximation of the Expectation Maximization and the Importance Sampling Estimation method. The Perl-Speaks-NONMEM program (version 4.8.1, Department of Pharmaceutical Biosciences, Uppsala University, Sweden) [33] and Pirana software (version 2.9.6, Pirana Software & Consulting BV) were used for model development and evaluation. Data processing and graphical analysis were conducted using R (version 4.0.0, http://cran.r-project.org, accessed on 14 April 2020) and R-Studio (version v2021.09.0+354).

Structural compartmental PK models (one-, two-, and three-compartment) parametrized in apparent volume of distribution and clearances were selected to characterize the time-course of daptomycin. Due to the high plasma protein-binding capacity of daptomycin, several protein-binding relationships were evaluated to account for the unbound and total concentrations, which are described as follows [34,35]:

- Linear binding model [36,37]
  (1) $$C_{total}=C_{free}+C_{bound}=C_{free}+K_{b}\cdot C_{free}$$
  where $C_{total}$ (mg/L) is the total daptomycin concentration, $C_{free}$ (mg/L) is the unbound daptomycin concentration, $C_{bound}$ (mg/L) is the bound daptomycin concentration and $K_{b}$ is the binding proportionality constant. This model assumes that $C_{bound}$ is directly proportional to $C_{free}$ with no saturable mechanism.
- Non-linear binding model with single site binding [38,39]:
  (2) $$C_{total}=C_{free}+B_{max}\cdot C_{free}/(K_{D}+C_{free})$$
  where $B_{max}$ (mg/L) represents the maximal binding capacity and $K_{D}$ (mg/L) is the equilibrium dissociation constant.
- Non-linear binding model with multiple site binding [38,40,41]
  (3) $$C_{total}=C_{free}+\sum^{\;}B_{max,i}\cdot C_{free}/(K_{D,i}+C_{free})$$
  where $B_{max,i}$ and $K_{D,i}$ represent the binding-dissociation parameters of the ith binding site of plasma proteins.

Inter-individual variability (IIV) associated with the PK model parameters was modeled exponentially, and the residual unexplained variability (RUV) was explored using the additive error model on the logarithmic scale. The significance of the non-diagonal elements of the Ω variance-covariance matrix was evaluated.

Once the base model was selected, a graphical exploration of the correlations between the PK parameters (when eta-shrinkage was less than 35%) [42] and covariates was performed. The continuous covariate included age, height, body weight, serum creatinine, creatinine clearance, and albumin. The categorical covariates were gender and statin co-administration. A numerical evaluation of the parameter-covariate relationship was performed manually in a univariate testing.

The model selection was based on the physiological and pharmacological rationale with the principle of parsimony [43]. A decrease in 3.84 points of the objective function value (OFV) provided by NONMEM^®^ between two nested models differing in one parameter was considered significant at the 5% level, together with the visual inspection of the goodness of fit (GOF) [44] and normalized prediction distribution error (NPDE) plots [45].

A model evaluation [46,47] was performed through prediction-corrected visual predictive checks (pc-VPC), [48] and a non-parametric bootstrap (*n* = 1000) was conducted to assess the statistical significance of the final parameter estimates.

Monte Carlo simulations (*n* = 10,000) were conducted assuming a log-normal distribution of PK parameters and a fixed combination of creatinine clearance (30, 60, 90 mL/min/1.73 m^2^) and body weight (50, 60, 70, 80, 90, and 100 kg). A 30-min intravenous infusion of daptomycin at 5–12 mg/kg every 12 (q12h) and 24 (q24h) hours or 10–17 mg/kg every 48 (q48h) hours were assumed.

The total area under the concentration curve (AUC) and unbound AUC (fAUC) were calculated after the first (AUC0-24) dose to represent the exposure at single dose conditions, respectively. Eight MIC values were considered: 0.25, 0.5, 0.75, 1, 1.5, 2, 3, and 4 mg/L.

A safety PK/PD index has been reported based on the trough levels (Cmin) of daptomycin ≥24.3 mg/L, which are associated with a 50% probability of creatine phosphokinase elevation [49]. The probability of Cmin ≥24.3 mg/L at 24 h was calculated for each scenario.

An optimal daptomycin dose selection was based on the probability of the target attainment (PTA) [50] ≥90% and ≤20% probability of achieving a Cmin ≥24.3 mg/L for each scenario.

## 3. Results

### 3.1. Patient Population and Study Design

A total of 46 patients, including 157 daptomycin observations, were included in the analysis, which were logarithmically transformed for model development. Table 1 summarizes the baseline demographic, treatment and clinical data of the cohort included in the dataset.

### 3.2. Population Pharmacokinetic Model

#### 3.2.1. Base Population PK Model

**Figure 1:**
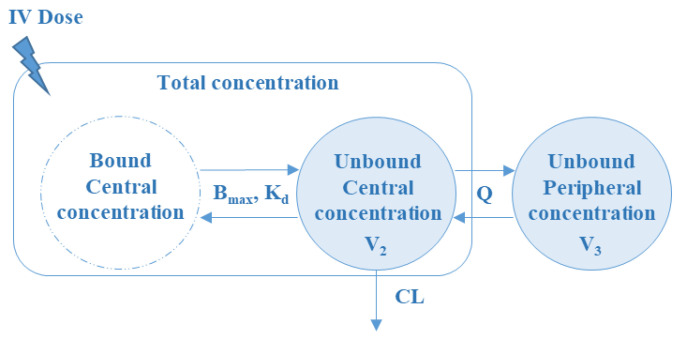
Schematic representation of the final population PK model. IV: intravenous; CL: clearance; V1: apparent volume of distribution of the unbound central compartment; Q: intercompartmental clearance; V2: apparent volume of distribution of the unbound peripheral compartment; Bmax: maximal binding capacity; KD: equilibrium dissociation constant.

A two-compartment model with first-order peripheral distribution and elimination kinetics using ordinary differential equations for unbound daptomycin concentrations was selected as the structural PK model (Figure 1).

The total concentrations of daptomycin were derived by assuming non-linear protein-binding kinetics (Equation (2)). Non-linear protein binding was found to significantly improve the description of the data compared with a linear implementation (ΔOFV = −23). The adequacy of the base model to capture the observed data can be found in Supplementary Material Figure S1 (GOF-NPDE) and Supplementary Material Figure S2 (pc-VPC).

#### 3.2.2. Final Population PK Model

The covariate analysis estimated a moderate but statistically significant relationship (∆OFV = −9.4) between CLCR and CL, using a power function (0.19) centered around the median value (Equation (4)):

(4) $$CL_{i}=CL\cdot {\left(\frac{CLCR_{i}}{92.8}\right)}^{0.19}$$

Additional continuous and categorical covariates were tested but did not result in a statistical (∆OFV < 3.84) reduction of the OFV.

The final population PK model shows a higher elimination clearance (CL = 6.98 L/h) and an apparent volume of distribution of the central (V1 = 0.95 L) compared to previous works using total daptomycin concentrations. This could be expected, since PK parameters are referred to unbound daptomycin concentrations. The peripheral distribution of unbound daptomycin represents a relevant PK process, based on the magnitude of the apparent volume of peripheral (V2) compartment and distribution (Q) clearance. A maximal binding capacity (Bmax) and KD of 160 and 3.56 mg/L, respectively, were estimated.

Moderate inter-individual random effects were identified for clearance (CL = 32%) and the apparent volume of the peripheral (V2 = 47%) compartment and the RUV was 22%. Table 2 lists the parameters of the base and final population PK models, together with the bootstrap results. All PK parameters were statistically significant, as the 95% confidence intervals (95% CI) from the bootstrap analysis did not include the null value.

**Figure 2:**
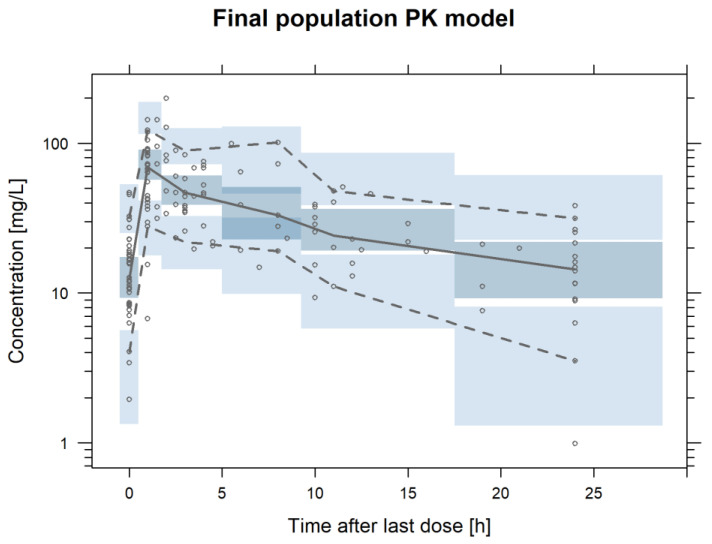
Prediction-Corrected Visual Predictive check of the final population pharmacokinetic model of daptomycin. Grey lines represent the 2.5th, 50th and 97.5th experimental percentiles. Blue shaded areas represent the 95% prediction interval of the 2.5th, 50th and 97.5th percentiles. Empty grey dots represent the experimental daptomycin observations.

Due to the different doses administered across the study, a prediction-corrected VPC was conducted, showing an adequate characterization of the longitudinal profiles of the median and the dispersion of the data of the final population PK model (Figure 2). Supplementary Material Figure S3 shows the GOF and NPDE plots of the final population PK model.

### 3.3. Population Pharmacokinetic/Pharmacodynamic Simulations and Optimal Dosage Selection

**Figure 3:**
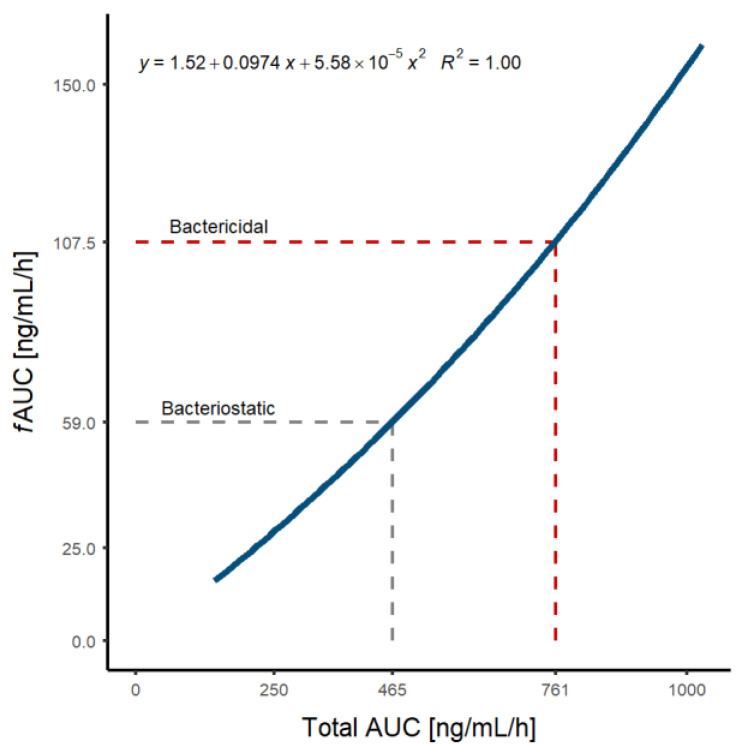
Predicted relationship between total and unbound (fAUC) AUC of daptomycin. Grey and red dotted lines represent the bacteriostatic and bactericidal AUC values, respectively, for Staphylococcus aureus for a MIC value of 1 mg/L.

Several authors have reported the AUC of daptomycin with a bacteriostatic (465 mg·h/L) [19] or bactericidal (666 [24,51,52] and 761 mg·h/L [19]) effect for an MIC equal to 1 mg/L. Based on the non-linear relationship (Figure 3) between total AUC and unbound AUC (fAUC), two efficacy PK/PD targets, defined as fAUC were considered: 59, and 107.5 mg·h/L.

**Figure 4:**
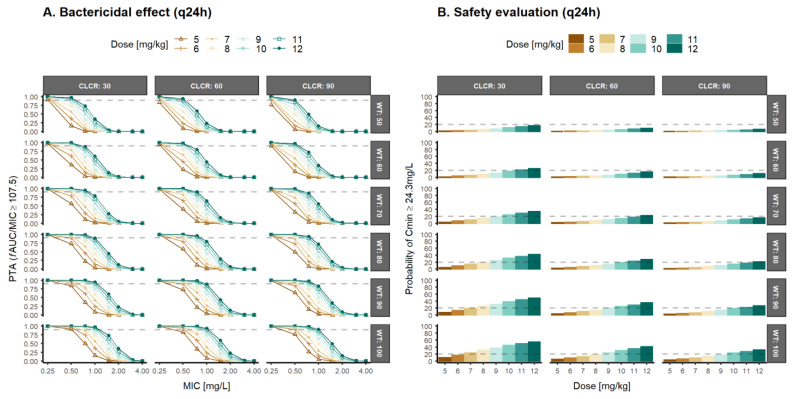
Probability of target attainment (PTA) after once daily (q24h) dose levels (5–12 mg/kg) of daptomycin in patients with different creatinine clearance (CLCR) and body weight (WT) for an (**A**) fAUC/MIC greater or equal to 107.5 (bactericidal effect), and (**B**) Predicted probability of achieving trough concentrations of total daptomycin greater or equal to 24.3 mg/L MIC: minimum inhibitory concentration; fAUC/MIC: unbound drug area under the concentration–time curve/minimum inhibitory concentration ratio.

PTA simulations after daptomycin 5–12 mg/kg q24h administration assuming a bactericidal effect (fAUC/MIC ≥107.5) for each combination of CLCR and body weight observed in this study, together with the probability of safety concerns (Cmin ≥24.3 mg/L), are depicted in Figure 4. PTA simulation after daptomycin 5–12 mg/kg q24h administration assuming a bacteriostatic effect (fAUC/MIC ≥59) is represented in Supplementary Material Figure S4.

A dose-dependent effect is observed throughout the dose levels evaluated, indicating that lower doses may be required in patients with a high body weight and decreased renal function. A bactericidal effect (fAUC/MIC ≥107.5) for Gram+ strains with MIC ≤0.5 mg/L (Figure 4) could be achieved with 5–12 mg/kg daily daptomycin in patients weighing 50–100 kg, but different dose ranges would be recommended based on renal function: 5–10 mg/kg (CLCR = 30 mL/min/1.73 m^2^), 6–11 mg/kg (CLCR = 60 mL/min/1.73 m^2^), and 6–12 mg/kg (CLCR = 90 mL/min/1.73 m^2^). Although higher dose levels have been proposed to achieve a bactericidal effect, especially in patients at the low body weight range and with less impaired renal function, the probability of trough daptomycin levels above 24.3 mg/L was below 20%, demonstrating the adequate benefit/risk balance of the daptomycin q24h dosing regimens proposed for Gram+ strains with MIC ≤0.5 mg/L. However, the results from the PTA analysis did not achieve the efficacy threshold when a bactericidal effect is expected for MRSA strains with MIC ≥1 mg/L (Figure 4). High dose levels (>9 mg/kg in patients with a high body weight and/or low CLCR) would be recommended, but relevant safety concerns may appear in those scenarios (Figure 4).

**Figure 5:**
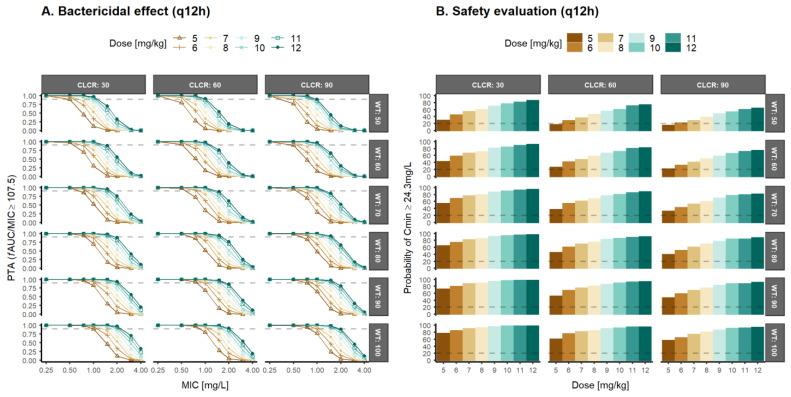
Probability of target attainment (PTA) after twice daily (q12h) dose levels (5–12 mg/kg) of daptomycin in patients with different creatinine clearance (CLCR) and body weight (WT) for an (**A**) fAUC/MIC greater or equal to 107.5 (bactericidal effect), and (**B**) Predicted probability of achieving trough concentrations of total daptomycin greater or equal to 24.3 mg/L MIC: minimum inhibitory concentration; fAUC/MIC: unbound drug area under the concentration–time curve/minimum inhibitory concentration ratio.

**Figure 6:**
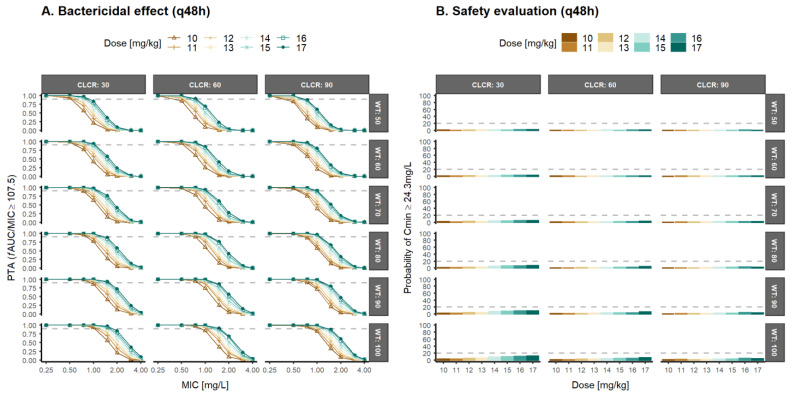
Probability of target attainment (PTA) after once every two days (q48h) dose levels (5–12 mg/kg) of daptomycin in patients with different creatinine clearance (CLCR) and body weight (WT) for an (**A**) fAUC/MIC greater or equal to 107.5 (bactericidal effect), and (**B**) Predicted probability of achieving trough concentrations of total daptomycin greater or equal to 24.3 mg/L MIC: minimum inhibitory concentration; fAUC/MIC: unbound drug area under the concentration–time curve/minimum inhibitory concentration ratio.

Therefore, alternative dosing regimens (q12h or q48h) were evaluated to select the optimal daptomycin dosing regimens with a bactericidal effect for MRSA strains (Figure 5 and Figure 6).

The global evaluation of the results obtained with the q12h regimen show high levels of PTA (≥90%) in the range of doses evaluated (5–12 mg/kg), although administration every 12 h increases by well over 20% the probability of daptomycin trough levels with risk of CPK elevation. The administration of 10–17 mg/kg q48h daptomycin allows reaching PTA ≥90% for MRSA Gram+ strains in patients with CLCR ≤30 mL/min/1.73 m^2^ or body weight ≥70 kg. For patients with CLCR ≥60 mL/min/1.73 m^2^ and body weight ≤60 kg, 17 mg/kg, q48h allows to achieve PTA 60–85% with a probability of trough levels less than 11%. In this sense, increasing the dose and spacing its administration at 48-h intervals would favor a satisfactory response rate in most subgroups evaluated, as well as a low probability of associated muscle toxicity. Supplementary Material Figures S5 and S6 depict the PTA simulations of daptomycin q12h and q48h assuming bacteriostatic effect.

Distribution of trough levels of daptomycin q12h, q24h or q48h obtained from the Monte Carlo simulation are represented in Supplementary Material Figures S7–S9.

## 4. Discussion

The selection of the optimal antibiotic treatment in patients with renal impairment represents one of the greatest challenges currently facing clinical teams. This is due to the increasingly frequent appearance of resistant strains (highly resistant S. aureus, methicillin-resistant S. aureus, and E. faecium, which leads to severe or life-threatening conditions [24,27,53]. The increase of PK and PD knowledge of daptomycin, together with the commitment to make a more rational and efficient decision-making process, has allowed for the development of model-informed strategies. This has become a fundamental tool for the selection of the optimal dosing regimen for daptomycin. In this sense, a two-compartment population PK model of the unbound fraction of daptomycin, based on a non-linear binding relationship with the total (observed) daptomycin concentrations, provides new insights into the PK properties of this drug to guide dose selection of daptomycin in patients with several degrees of renal impairment and body weight ranges. In addition, the model can inform an optimal PK sampling scenario to evaluate individual safety and efficacy in daptomycin TDM.

The structural PK parameters estimated in this study are referred to the predicted unbound fraction of daptomycin. If a 10% unbound fraction of daptomycin is assumed, the CL (CL = CLu × fu) and Q (Q = Qu × fu) of total daptomycin concentrations are in agreement with the previously reported values [25]. Daptomycin is highly (90%) reversibly bound to albumin and α-glycoprotein [24,53]. The variation of the latter, especially in patients with renal impairment, may affect the unbound daptomycin concentration. Therefore, the characterization of the unbound fraction of daptomycin is of special relevance, since it is the active form of the drug that is able to exert the pharmacological response [25]. Thus, a PK/PD target based on unbound concentrations is more suitable for a target attainment assessment [35]. In previous works, the dose selection was based on the total daptomycin levels or assuming a linear unbound fraction of 10% for PK/PD indexes, which could lead to biased conclusions due to differences in protein binding and the apparent volume of distribution that have been reported in patients with impaired renal function [3,5]. In this work, we have developed a population PK model of daptomycin, which considers a non-linear binding kinetics to human plasma proteins, that allows to predict free daptomycin concentrations from total daptomycin values. Although albumin levels could not be related to the binding mechanism, the results show a high binding affinity of daptomycin to plasma proteins (K_D_ = 1.96 mg/L), with a maximal binding capacity reached at 160 mg/L. The maximal binding capacity reflects the concentration of the binding protein and the number of available binding sites [54]. Based on the average albumin concentration (2.95 g/L), it seems reasonable to suggest that albumin is the primary binding site of daptomycin in a 1:1 molar relationship. At the range of usual concentrations of daptomycin, saturable binding conditions appear. This leads to a more than proportional increase in fAUC as AUC increases, that, if not considered, may affect the selection of an optimal dosage regimen.

A concentration-dependent antibiotic effectiveness of daptomycin was characterized that is best correlated to AUC/MIC [52]. The selected AUC/MIC values (465 and 761) represent the most favorable and restrictive scenarios. Their corresponding fAUC/MIC were derived (Figure 3) that allows the covering of a PK/PD range in order to explore the selection of the dosage regimen according to the desired therapeutic objective. To reach a bacteriostatic effect (Table 3), a dose recommendation of 5–7 mg/kg for Gram-positive strains (MIC ≤0.5 mg/L) in patients with body weight ≥50 kg and 5–11 mg/kg for MRSA strains once daily of daptomycin in ≥60 kg is in agreement with previous reports [55,56,57,58,59,60,61,62,63].

The schedule optimization with bactericidal effect for Gram-positive strains with MIC ≤0.5 mg/L, suggested that safe and efficacious 5–12 mg/kg once daily daptomycin dosing regimens can be proposed in patients from 50 to 100 kg and different degrees of CLCR (Table 3).

Given the appearance of more resistant strains (MIC ≥1 mg/L) in patients with normal renal function, PTA results (Figure 4) suggested daily regimens of daptomycin between 9 and 12 mg/kg in patients with a high body weight (≥80 kg) or moderate renal impairment (CLCR = 30 mL/min/1.73 m^2^) resulted in a probability of trough concentration ≥24.3 mg/L higher than 20%. This is correlated with CPK elevations and, therefore, muscle toxicity [1,2,3,5] (Figure 5). These results are in agreement with previous works, suggesting that higher dose levels than the approved ones of daptomycin may be required [19,21,49,64,65,66]. Therefore, it is evident for the need to explore more frequent schemes with lower dosage levels in order to guarantee optimal PTA levels and safety. According to the results from Table 3, the treatment of infections with MRSA Gram-positive strains requires higher dose levels every 48 h in order to achieve adequate efficacy (PTA > 90%) and safety (Cmin > 24.3 mg/L) objectives. Intensive dosing regimens (q12h) resulted in a high probability of trough concentrations of daptomycin above the safety threshold (Figure 6). Alternatively, it may be necessary to evaluate fixed-dose schemes that allow reducing the differences in the magnitude of doses associated with body weight. The risk of over-exposure in low weight patients is minor, and the under-exposure associated with the fixed dose that would lead to risks of inefficacy in heavy patients could be of little clinical relevance based on the PTA results.

Among the main limitations of this analysis, it is important to highlight the absence of observations of free daptomycin that would allow us to identify with greater certainty the degree of binding to plasma proteins of daptomycin in vivo. However, similar structural PK models have been used to characterize drug binding kinetics based on total concentrations [67,68]. On the other hand, a greater number of observations per individual, as well as the availability of patients with severe renal insufficiency, would be of great relevance for the characterization of the influence of CLCR on clearance and, with it, the design of dosage schemes in this subgroup of patients. In addition, the lack of women (7%) enrolled in this study due to the study design could limit the evaluation of the gender effect on the PK parameters. Therefore, additional studies, including a more balanced distribution of sub-groups of interest, are encouraged to evaluate their dosing recommendations of daptomycin.

## 5. Conclusions

In conclusion, the two-compartment population PK model has first allowed us to characterize the non-linear binding kinetics of daptomycin in patients with renal impairment. This served to evaluate the impact of different dosing regimens on the efficacy and safety outcomes of daptomycin treatment, based on the unbound exposure of daptomycin and individual patient characteristics.

## Figures and Tables

**Table 1:** Summary of study design characteristics.

Characteristics		46 Patients Median (IQR)/*n* (%)
Demographics	Sex (Male) (*n*, %)	43 (93%)
	Age (years)	68 (59–81)
	Body weight (kg)	75 (65–85)
	Height (m)	1.7 (1.6–1.7)
	BMI (Kg/m^2^)	25.9 (23.4–31.1)
Treatment	Dose (mg)	675 (500–765)
	Dose per kilogram (mg/kg)	9.1 (7.5–10.0)
	Treatment duration (days)	11 (7–15)
Clinical data	Serum albumin (g/dL)	2.9 (2.4–3.4)
	Serum protein (g/dL)	6.0 (5.0–6.4)
	Serum cretinine (g/dL)	0.9 (0.6–1.3)
	Creatinine clearance (mL/min/1.73 m^2^)	93 (50–136)
	Renal function	
	>90 mL/min/1.73 m^2^	16 (34.9%)
	60–89 mL/min/1.73 m^2^	13 (28.2%)
	30–59 mL/min/1.73 m^2^	14 (30.4%)
	15–29 mL/min/1.73 m^2^	3 (6.5%)
	<15 mL/min/1.73 m^2^	0 (0%)
Pathogenic micro-organism (36/46)	*S. aureus*	17 (47.2%)
	*S. epidermidis*	12 (33.3%)
	*S. hominis*	2 (5.6%)
	*E. fecalis*	2 (5.6%)
	*S. sacrophyticus*	2 (5.6%)
	*S. lugdunensis*	1 (2.7%)
	MIC micro-organism	0.5 (0.25, 0.5)

IQR: interquartile range, *n*: number of individuals.

**Table 2:** Final population pharmacokinetic parameters of daptomycin in patients with normal renal function and renal impairment.

	Population PK Model Estimates			Bootstrap Results		
	Value	RSE (%)	Shrinkage (%)	Median	RSE * (%)	95%CI
***Fixed-Effect***						
CL (L/h)	6.98	14		7.01	15	[6.63–7.44]
V_1_ (L)	0.95	9		0.97	10	[0.92–1.09]
Q (L/h)	1.96	21		1.93	19	[1.43–2.48]
V_2_ (L/h)	21	19		20.5	21	[19.3–22.1]
B_max_ (mg/L)	160	26		157	24	[129–183]
K_D_ (mg/L)	3.56	15		3.61	12	[3.17–3.93]
CrCl on CL	0.19	12		0.19	13	[0.18–0.22]
***Inter-individual variability***						
CL (%)	32	11	12	33	10	[21–42]
V_2_ (%)	47	23	17	46	24	[52–94]
***Residual unexplained variability***						
Additive on Log-scale (%)	22	8	5	21	8	[18–24]

* RSE: relative standard error.

**Table 3:** Daptomycin dosing schedules proposed based on the MIC, renal function and body weight of the patient to achieve bactericidal effect.

	Moderate Renal Impairment (CL_CR_ = 30 mL/min/1.73 m^2^)	Mild Renal Impairment (CL_CR_ = 60 mL/min/1.73 m^2^)	Normal Renal Function (CL_CR_ = 90 mL/min/1.73 m^2^)	Body Weight
**MIC ≤ 0.5 mg/L**	10 mg/kg q24h	11 mg/kg q24h	12 mg/kg q24h	**50 kg**
	9 mg/kg q24h	10 mg/kg q24h	10 mg/kg q24h	**60 kg**
	8 mg/kg q24h	9 mg/kg q24h	9 mg/kg q24h	**70 kg**
	7 mg/kg q24h	7 mg/kg q24h	8 mg/kg q24h	**80 kg**
	6 mg/kg q24h	7 mg/kg q24h	7 mg/kg q24h	**90 kg**
	5 mg/kg q24h	6 mg/kg q24h	6 mg/kg q24h	**100 kg**
**MIC ≤ 1 mg/L**	17 * mg/kg q48h	17 * mg/kg q48h	17 * mg/kg q48h	**50 kg**
	16 mg/kg q48h	17 * mg/kg q48h	17 * mg/kg q48h	**60 kg**
	14 mg/kg q48h	16 mg/kg q48h	17 mg/kg q48h	**70 kg**
	12 mg/kg q48h	14 mg/kg q48h	15 mg/kg q48h	**80 kg**
	11 mg/kg q48h	12 mg/kg q48h	13 mg/kg q48h	**90 kg**
	10 mg/kg q48h	11 mg/kg q48h	12 mg/kg q48h	**100 kg**

MIC: minimum inhibitory concentration; CL_CR_: creatinine clearance; * PTA results <90%.

## Data Availability

Not applicable.

## Supplementary Materials

The following supporting information can be downloaded at: https://www.mdpi.com/article/10.3390/pharmaceutics14102226/s1, Figure S1: Goodness-of-fit plots of the base population pharmacokinetic model of daptomycin; Figure S2: VPC of the base population pharmacokinetic model of daptomycin; Figure S3: Goodness-of-fit plots of the final population pharmacokinetic model of daptomycin; Figure S4: PTA after once daily (q24h) dose levels (5–12 mg/kg) of daptomycin in patients with different CL_CR_ and WT for an (**A**) *f*AUC/MIC greater or equal to 59 (bacteriostatic effect), and (**B**) Predicted probability of achieving trough concentrations of total daptomycin greater or equal to 24.3 mg/L; Figure S5: PTA after twice daily (q12h) dose levels (5–12 mg/kg) of daptomycin in patients with different CL_CR_ and WT for an (**A**) *f*AUC/MIC greater or equal to 59 (bacteriostatic effect), and (**B**) Predicted probability of achieving trough concentrations of total daptomycin greater or equal to 24.3 mg/L; Figure S6: PTA after once every other day (q48h) dose levels (5–12 mg/kg) of daptomycin in patients with different CL_CR_ and WT for an (**A**) *f*AUC/MIC greater or equal to 59 (bacteriostatic effect), and (**B**) Predicted probability of achieving trough concentrations of total daptomycin greater or equal to 24.3 mg/L; Figure S7: Daptomycin trough concentrations (Cmin) after single dose levels (5–12 mg/kg) of daptomycin every 24 h in patients with different CL_CR_) and WT; Figure S8: Daptomycin trough concentrations (Cmin) after single dose levels (5–12 mg/kg) of daptomycin every 12 h in patients with different CL_CR_ and WT; Figure S9: Daptomycin trough concentrations (Cmin) after single dose levels (10–17 mg/kg) of daptomycin every 48 h in patients with different CL_CR_ and WT.
