# Supplementary material for: Population Pharmacokinetic/Pharmacodynamic Modelling of Daptomycin for Schedule Optimization in Patients with Renal Impairment
Source: Pharmaceutics. 2022 Oct 18;14(10):2226. doi: 10.3390/pharmaceutics14102226 (PMC9607246; doi:10.3390/pharmaceutics14102226)
Supplement: Supplementary file 1 [file pharmaceutics-14-02226-s001.zip › pharmaceutics-1909648-supplementary.pdf]

pharmaceutics

MDPI

Supplementary Materials: Population Pharmacokinetic/Pharmacodynamic Modelling of Daptomycin for Schedule Optimization in Patients with Renal Impairment

Teresa García-Martínez, María Dolores Bellés-Medall , Maria García-Cremades , Raúl Ferrando-Piqueres , Victor Mangas-Sanjuán and Matilde Merino-Sanjuan

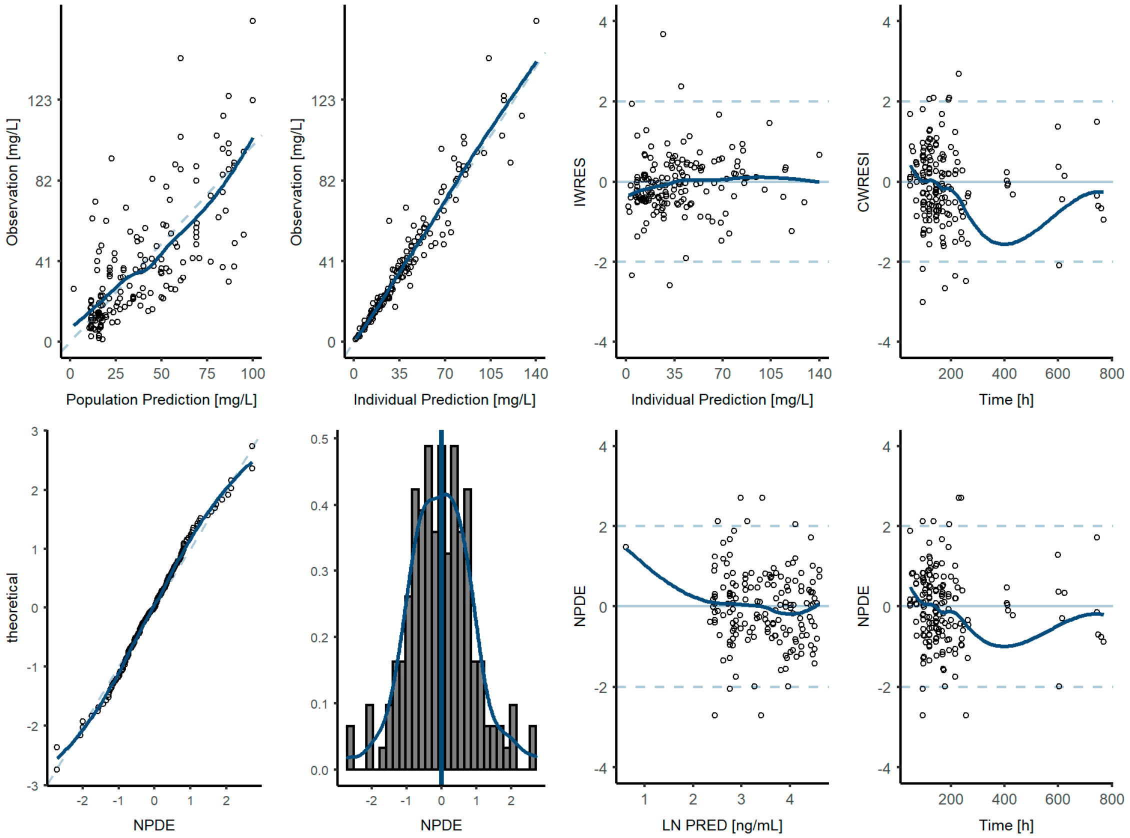

Figure S1. Goodness-of-fit plots of the base population pharmacokinetic model of daptomycin. NPDE: normalized prediction distribution error; LN: log-transformed observations.

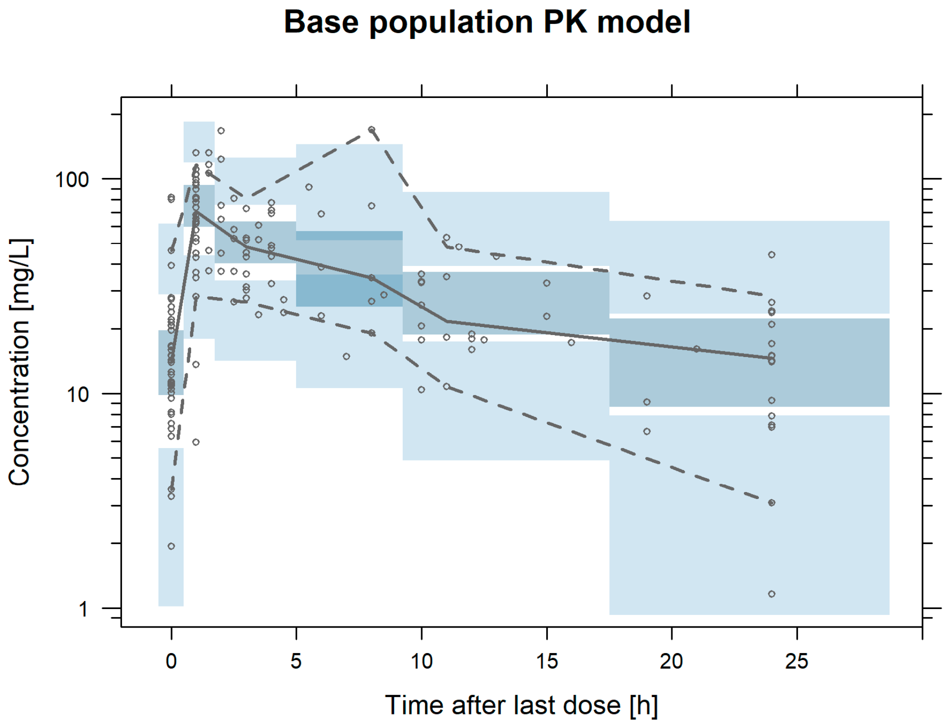

Figure S2. Visual Predictive check of the base population pharmacokinetic model of daptomycin. Grey lines represent the 2.5th, 50th and 97.5th experimental percentiles. Blue shaded areas represent the 95% prediction interval of the 2.5th, 50th and 97.5th percentiles. Empty grey dots represent the experimental daptomycin observations.

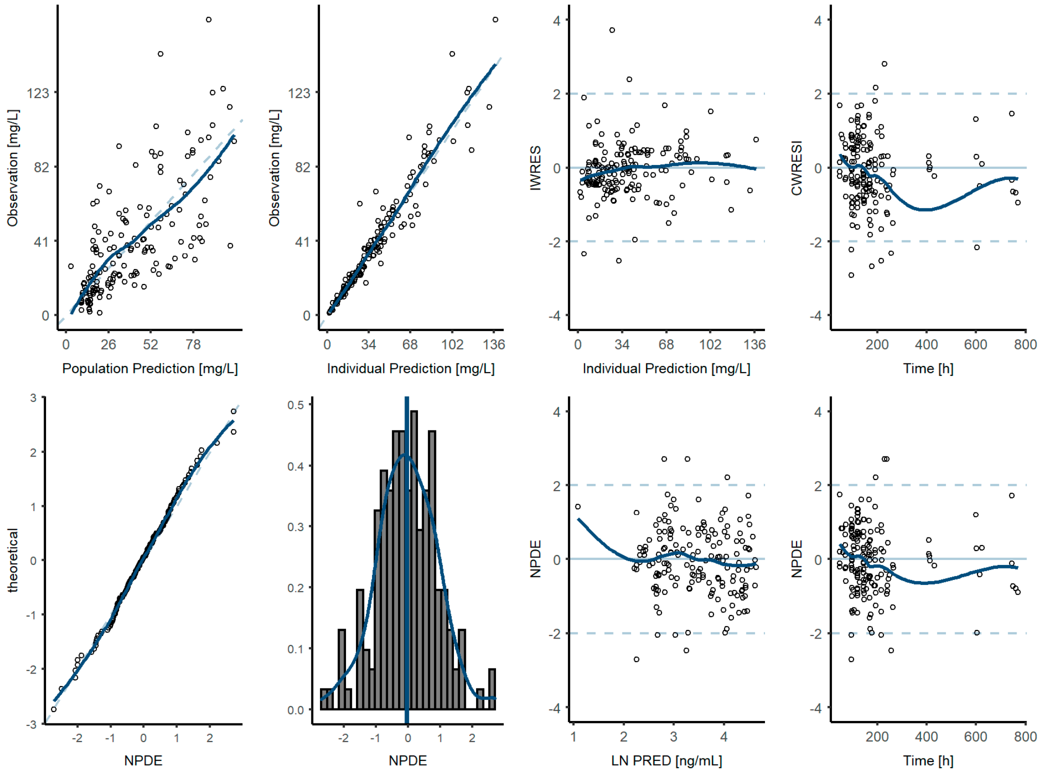

Figure S3. Goodness-of-fit plots of the final population pharmacokinetic model of daptomycin. NPDE: normalized prediction distribution error; LN: log-transformed observations.

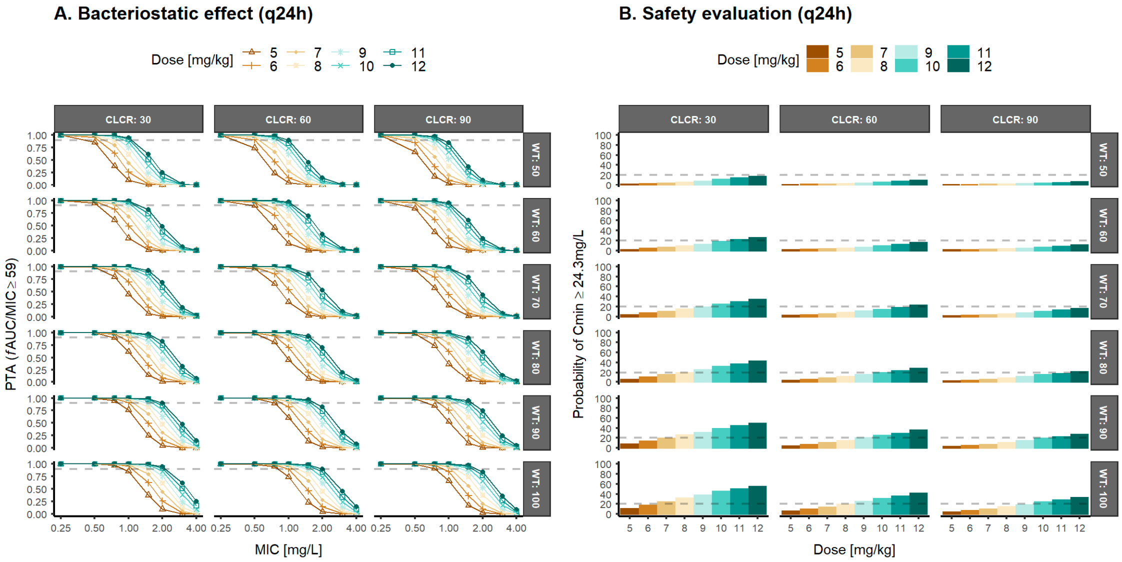

Figure S4. Probability of target attainment (PTA) after once daily (q24h) dose levels (5-12 mg/kg) of daptomycin in patients with different creatinine clearance (CLCR) and body weight (WT) for an (**A**) *f*AUC/MIC greater or equal to 59 (bacteriostatic effect), and (**B**) Predicted probability of achieving through concentrations of total daptomycin greater or equal to 24.3 mg/L MIC: minimum inhibitory concentration; *f*AUC/MIC: unbound drug area under the concentration-time curve/minimum inhibitory concentration ratio.

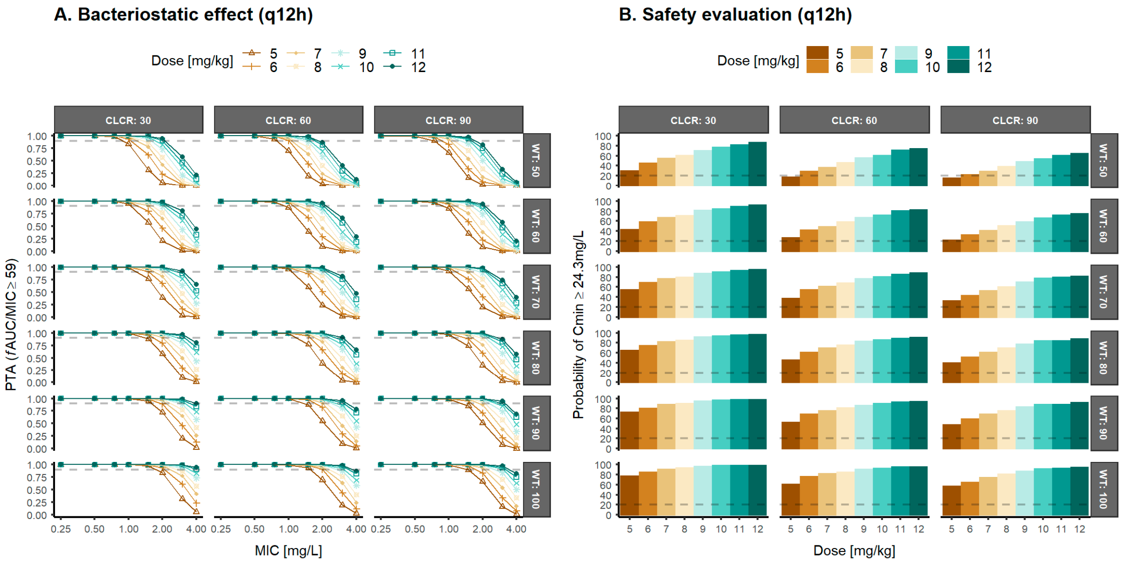

Figure S5. Probability of target attainment (PTA) after twice daily (q12h) dose levels (5-12 mg/kg) of daptomycin in patients with different creatinine clearance (CLCR) and body weight (WT) for an (**A**) *f*AUC/MIC greater or equal to 59 (bacteriostatic effect), and (**B**) Predicted probability of achieving through concentrations of total daptomycin greater or equal to 24.3 mg/L MIC: minimum inhibitory concentration; *f*AUC/MIC: unbound drug area under the concentration-time curve/minimum inhibitory concentration ratio.

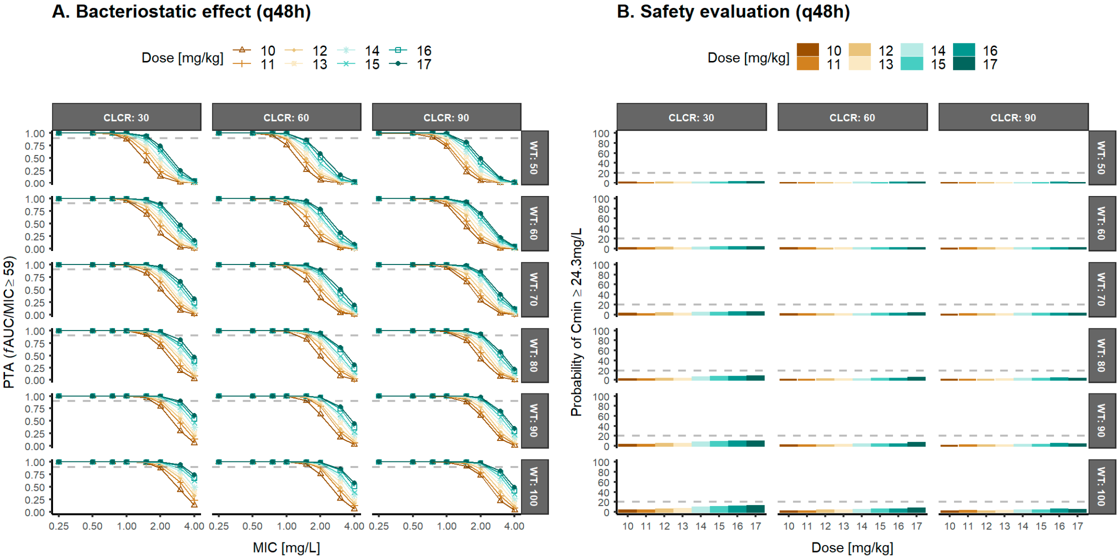

Figure S6. Probability of target attainment (PTA) after once every other day (q48h) dose levels (5-12 mg/kg) of daptomycin in patients with different creatinine clearance (CL_{CR}) and body weight (WT) for an (**A**) *f*AUC/MIC greater or equal to 59 (bacteriostatic effect), and (**B**) Predicted probability of achieving through concentrations of total daptomycin greater or equal to 24.3 mg/L MIC: minimum inhibitory concentration; *f*AUC/MIC: unbound drug area under the concentration-time curve/minimum inhibitory concentration ratio.

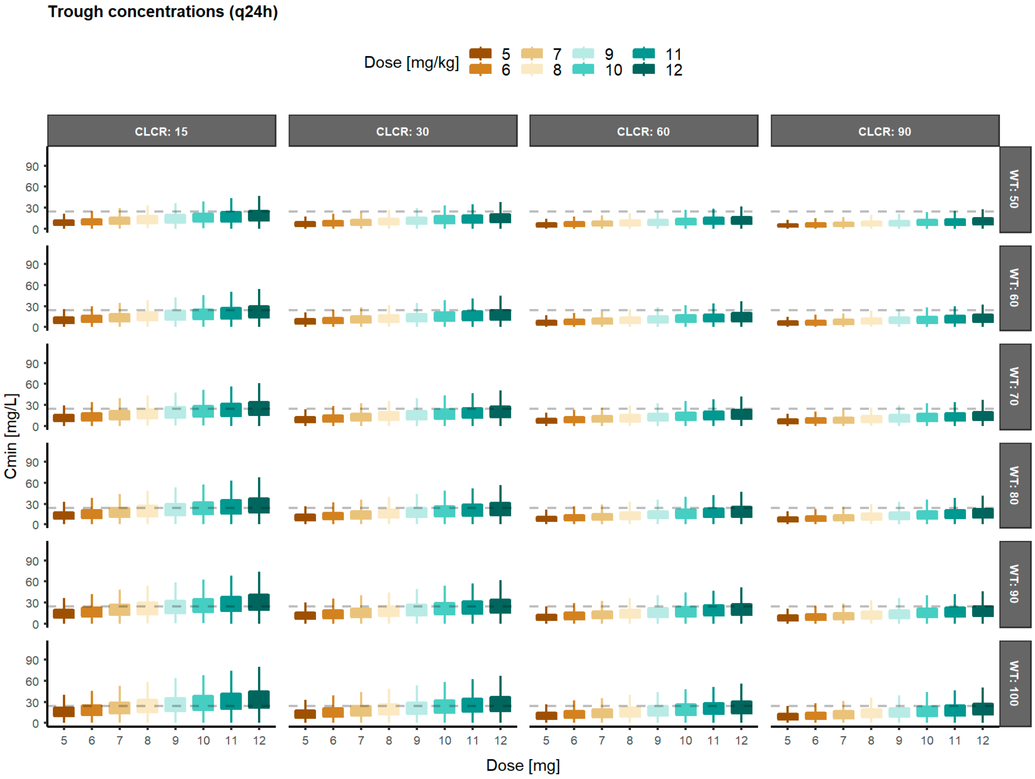

Figure S7. Daptomycin trough concentrations (Cmin) after single dose levels (5-12 mg/kg) of daptomycin every 24 hours in patients with different creatinine clearance (CL_{CR}) and body weight (WT). Grey dashed line represents the safety threshold of 24.3 mg/L of daptomycin. Boxplot represents the 25th and 75th percentiles (IQR) of the overall distribution and whiskers include the 1.5*IQR.

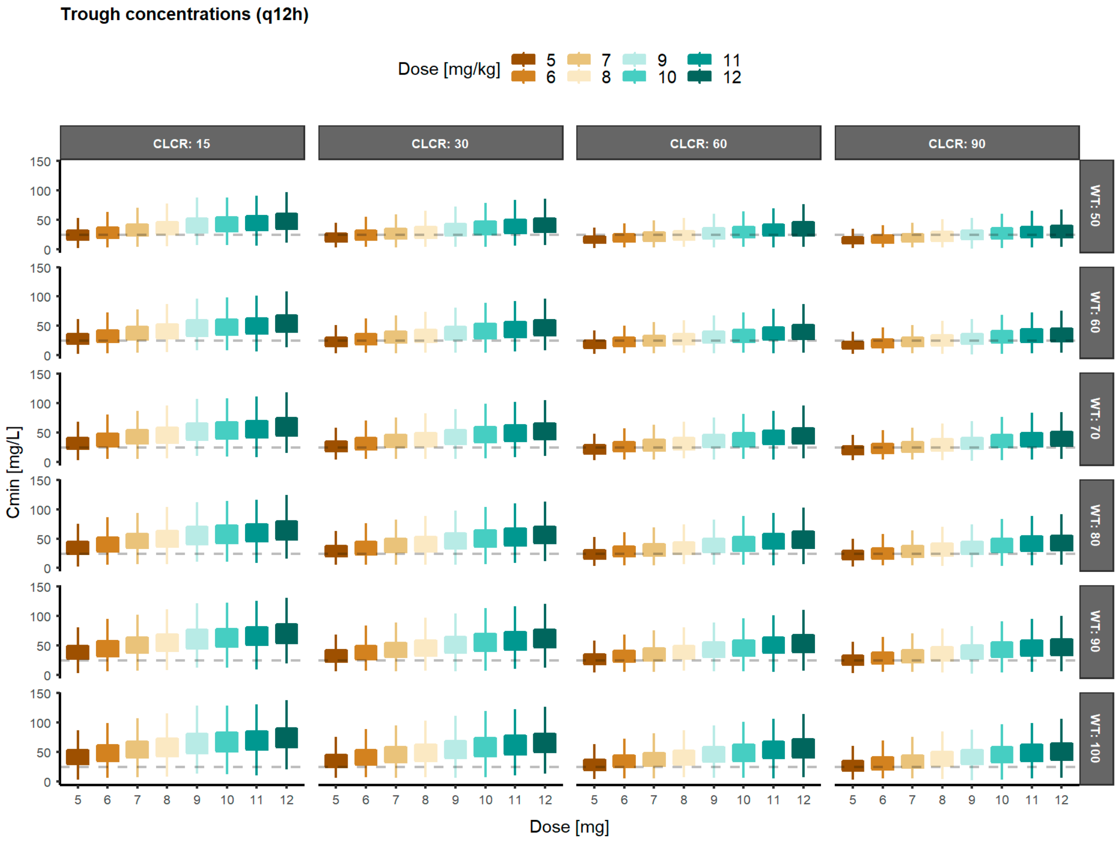

Figure S8. Daptomycin trough concentrations (Cmin) after single dose levels (5-12 mg/kg) of daptomycin every 12 hours in patients with different creatinine clearance (CL_{CR}) and body weight (WT). Grey dashed line represents the safety threshold of 24.3 mg/L of daptomycin. Boxplot represents the 25th and 75th percentiles (IQR) of the overall distribution and whiskers include the 1.5*IQR.

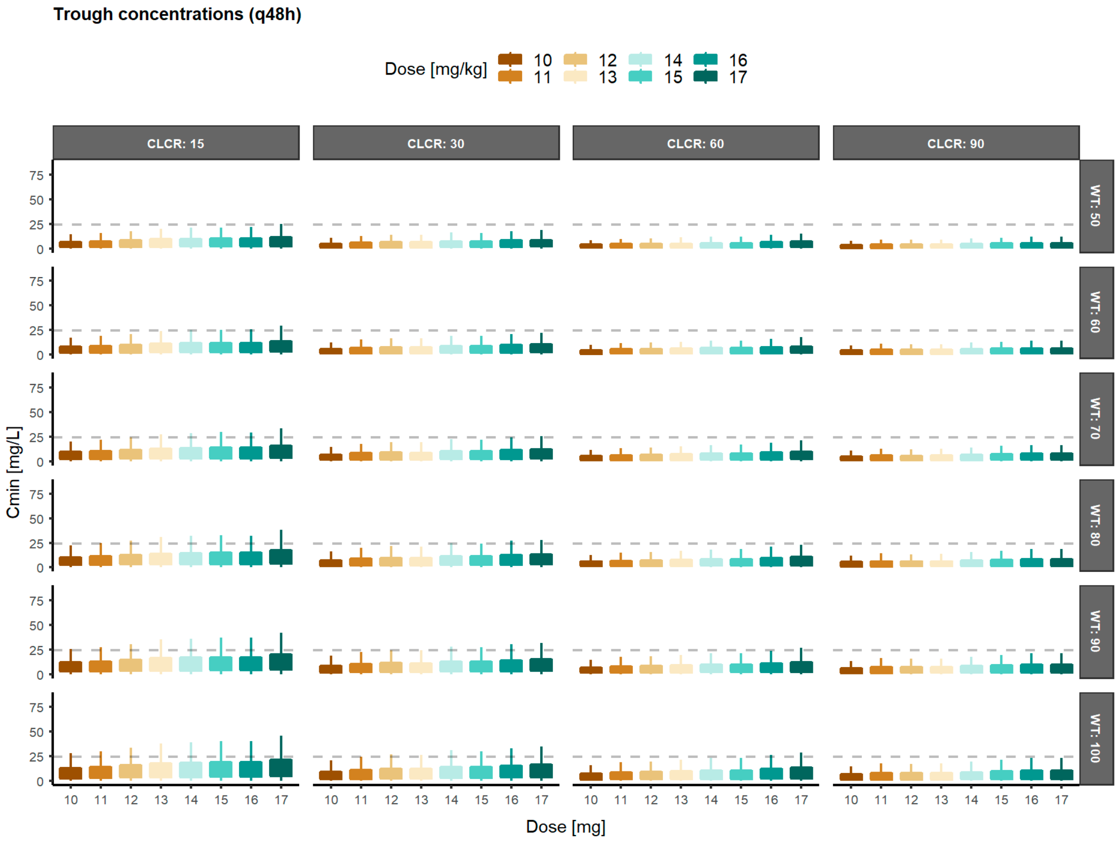

Figure S9. Daptomycin trough concentrations (Cmin) after single dose levels (10-17 mg/kg) of daptomycin every 48 hours in patients with different creatinine clearance (CL_{CR}) and body weight (WT). Grey dashed line represents the safety threshold of 24.3 mg/L of daptomycin. Boxplot represents the 25th and 75th percentiles (IQR) of the overall distribution and whiskers include the 1.5*IQR.
